# Towards facing uncertainties in biofuel supply chain networks: a systematic literature review

**DOI:** 10.1007/s11356-023-29331-w

**Published:** 2023-09-02

**Authors:** Farhad Habibi, Ripon K. Chakrabortty, Alireza Abbasi

**Affiliations:** School of Systems and Computing, UNSW Canberra, Canberra, ACT-2610 Australia

**Keywords:** Literature review, Biomass supply chain, Risk management, Resilient supply chain, PRISMA, Renewable energy

## Abstract

Biofuel supply chains (BSCs) face diverse uncertainties that pose serious challenges. This has led to an expanding body of research focused on studying these challenges. Hence, there is a growing need for a comprehensive review that summarizes the current studies, identifies their limitations, and provides essential advancements to support scholars in the field. To overcome these limitations, this research aims to provide insights into managing uncertainties in BSCs. The review utilizes the Systematic Reviews and Meta-Analyses (PRISMA) method, identifying 205 papers for analysis. This study encompasses three key tasks: first, it analyses the general information of the shortlisted papers. Second, it discusses existing methodologies and their limitations in addressing uncertainties. Lastly, it identifies critical research gaps and potential future directions. One notable gap involves the underutilization of machine learning techniques, which show potential for risk identification, resilient planning, demand prediction, and parameter estimations in BSCs but have received limited attention. Another area for investigation is the potential of agent-based simulation, which can contribute to analysing resilient policies, evaluating resilience, predicting parameters, and assessing the impact of emerging technologies on BSC resilience in the twenty-first century. Additionally, the study identifies the omission of various realistic assumptions, such as backward flow, lateral transshipments, and ripple effects in BSC. This study highlights the complexity of managing uncertainties in BSCs and emphasizes the need for further research and attention. It contributes to policymakers’ understanding of uncertain sources and suitable approaches while inspiring researchers to address limitations and generate breakthrough ideas in managing BSC uncertainties.

## Introduction

Energy plays a very prominent role in society, exerting direct and indirect influence over key sectors, including the economy, industry, and transportation (Lin and Chen 2020). Global energy consumption and demand are rising primarily due to the increasing human population, lifestyle changes, and fast industrial growth (Asif et al. 2023b). Environmental issues such as air pollution, greenhouse gas (GHG) emissions, and global warming have led to an increase in the use of sustainable and renewable energy sources (Asif et al. 2023a). Consequently, environmental-friendly energy sources, including solar, wind, and bioenergy, have received great attention over the last few years to address existing energy challenges (Ali et al. 2023). Biofuel has been considered a suitable alternative to fossil fuels due to having low lifecycle GHG emission, low price, the possibility of large-scale production, and widespread application in all sectors (Abbasi et al. 2021).

Biofuels are made from biomass (feedstock) and comprise gas, liquid, and solid fuels. Biofuels and their production technologies are generally classified into four generations based on the type of feedstock utilized in their production process (Mat Aron et al. 2020). First-generation biofuels, as the most generated type, are retained from edible biomass such as corn, wheat, barley, and sugarcane. Since this generation is threatening food security, the second generation emerged to use non-edible or lignocellulosic biomass, such as corn stover, switchgrass, and woody crops, as the feedstock (Bairamzadeh et al. 2018). The third-generation biomass uses microalgae biomass as the feedstock to produce biofuel. Microalgae grow faster and do not need large land or arable to flourish compared with other plant types. As a result, there is no rivalry between the agricultural sector, animal habitat, and human housing (Habibi et al. 2018; Zerafati et al. 2022). Genetically manipulated microalgae are used as biomass in fourth-generation biofuel. In this category, sophisticated technology produces modified microalgae that can capture significant carbon dioxide, boost biofuel yield, and grow wastewater (Mat Aron et al. 2020).

**Fig. 1:**
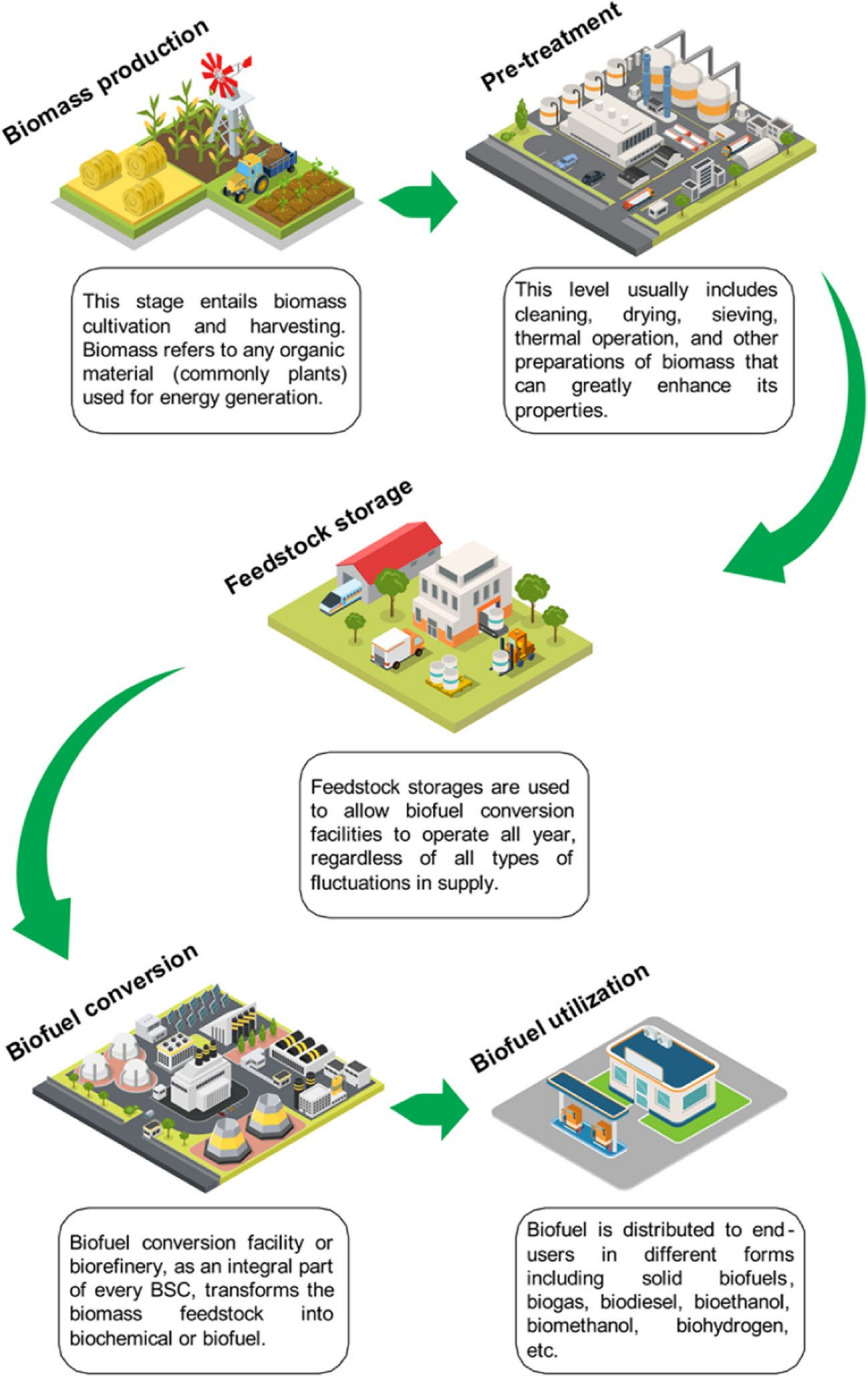
The general operations of biofuel production

The biofuel supply chain (BSC) typically encompasses multiple operations, ranging from biomass production and pre-treatment to storage, transfer to bio-refineries, and distribution to end users. The order and specific details of each operation are depicted in Fig. 1.

These operations can be carried out either in one centralized facility or several decentralized facilities (Ying et al. 2020). As a result, designing the whole BSC from a system viewpoint is quite complex and creates fresh challenges for decision-makers (Lan et al. 2020). The complexity of BSC is thought to stem from the interdependence among the modules depicted in Fig. 1 that are required for an uninterrupted supply of biomass. Furthermore, the biofuel supply chain (BSC) is regarded as being more vulnerable to risks when compared to conventional industrial supply chain networks (Mohammadi et al. 2023). The main reasons are the substantial uncertainties and disruptions associated with different parameters, including feedstock (Habibi et al. 2018), conversion processes (Lo et al. 2023; Sengupta and Pal 2021), pricing information (Lo et al. 2023), and demand rate (Asadi et al. 2018). This means that the factors that govern the BSC are not only too many but also highly uncertain. By way of illustration, consider the algae BSC, where the production rate of algae (as the feedstock) highly depends on sunny days, which may have negligible effects on other typical supply chains such as automotive or pharmaceutical industries. As another example, the feedstock and biofuel prices both heavily rely on the crude oil price, which is ever-changing (Habib et al. 2021). This shows the high depth of uncertainty in the parameters of a BSC. Due to the communities’ dependence on energy, energy supply chain networks are more prone to disruptions caused by targeted attacks, such as cyberattacks, sabotages, and vandalisms (Wang et al. 2023). The broad-scale adoption of biofuel systems remains limited due to the challenges with biomass feedstock (e.g. substantial fluctuations in biomass quality, quantity, and timelines) (El-Sheekh et al. 2019), conversion process (e.g. operational disruptions), and supply chain networks (e.g. serious risks and complexities) (Liao and Yao 2021). The root cause of this limitation is the challenges with uncertainties and reliability of biofuel systems, as mentioned by Liao and Yao (2021).

On one hand, uncertainties and disruptions are unavoidable in today’s business landscape, while on the other hand, organizations often face challenges in effectively recovering and resuming their operations following such disruptions. Proposing deterministic models and ignoring the effects of uncertainties and disruptions when planning in this area not only does not overcome the mentioned barriers but can also lead to infeasible design or sub-optimal outcomes (Vincent et al. 2023). Therefore, all of these have encouraged researchers to put their best effort into coming up with breakthrough ideas in handling uncertainties and disruptions in BSC through various methodologies such as designing the resilient biofuel system, predicting uncertainties, controlling the BSC during disruptions, and addressing detrimental effects. These methods empower BSC facilities to efficiently respond to, adapt, and recover from disruptions to satisfy the system’s goals and ensure effective performance to satisfy the consumers’ biofuel needs. If these criteria are met, the network can be called resilience, and biofuel supply chain resilience (BSCR) will be achieved.

### Study background

Given the numerous scientific endeavours that addressed uncertainties in BSCs, conducting a literature review becomes imperative to introduce, summarize, and categorize these methodologies. By doing so, the review aims to shed light on the existing challenges, pave the way for future research, and provide a comprehensive overview of the advancements made in this field. However, the BSC literature lacks such review studies. Several review papers are available that cover various aspects of BSC, including different decision types in biofuel and petroleum-based fuel (An et al. 2011), models of lignocellulosic biomass supply chains (Albashabsheh and Stamm 2021; Makepa et al. 2023; Santos et al. 2019; Verma et al. 2017), forest fuel networks (Strandgard et al. 2019; Wolfsmayr and Rauch 2014), sustainability concepts (Awudu and Zhang 2012; Hong et al. 2016), modelling uncertainties and decision-making levels (Awudu and Zhang 2012), microalgae-to-biofuel supply chain (Abbasi et al. 2021), quantitative models (Agustina et al. 2018; Ba et al. 2016; Fichtner and Meyr 2017; Ghaderi et al. 2016; Sun and Fan 2020; Zahraee et al. 2020; Zandi Atashbar et al. 2018), operational management research techniques (Ying et al. 2020), applications of artificial intelligence (AI) to bioenergy systems (Liao and Yao 2021), forest biomass supply chain resilience (Dashtpeyma and Ghodsi 2021), equipment used for biofuel production (Martinez-Valencia et al. 2021), biomass transportation and logistics (Ko et al. 2018), and quantitative and analytical risk models (Fahimnia et al. 2015). However, a few of these review studies examined the uncertainties in BSC, all of which have at least one of the following shortcomings:

- Some studies, such as Makepa et al. (2023), Dashtpeyma and Ghodsi (2021), and Abbasi et al. (2021), have focused solely on specific types of biofuel generation, such as lignocellulosic, forest, or microalgae biomass supply chains, thereby limiting their scope.
- Certain reviews have only touched upon certain uncertainties, omitting a comprehensive examination of uncertain sources and methodologies. For example, Sun and Fan (2020) primarily discuss common sources of uncertainties without delving into detailed information.
- Outdated studies, such as Awudu and Zhang (2012), fail to account for recent advancements, thus not providing researchers with up-to-date research directions and breakthrough insights (Awudu and Zhang 2012).
- While some book chapters discussed uncertainties in BSC (Pishvaee et al. 2021a) and modelling approaches to face uncertainties (Pishvaee et al. 2021b), they lack a thorough analysis of the limitations of existing studies and fail to offer specific suggestions for future research.

Table 1 compares the existing literature review papers in the BSC field with the current study.

**Table 1:** The summary of the review papers in the BSC field that discussed uncertainty

Review articles	Biofuel generation				Cover the last 5-year publications	Review of uncertainty sources		Review of methodologies to face uncertainties		Discussing limitations of existing methodologies
	1st	2nd	3rd	4th		Brief	In-depth	Brief	In-depth	
Awudu and Zhang (2012)	✓	✓	✓	✓			✓		✓	✓
Cobuloglu and Büyüktahtakin (2014)		✓						✓		
Yue et al. (2014)	✓	✓	✓	✓			✓	✓		
Ghaderi et al. (2016)	✓	✓	✓	✓			✓	✓		
Ba et al. (2016)	✓	✓	✓	✓				✓		
Santos et al. (2019)		✓						✓		
Sun and Fan (2020)	✓	✓	✓	✓		✓		✓		
Pishvaee et al. (2020a)	✓	✓	✓	✓	✓		✓			
Pishvaee et al. (2020b)	✓	✓	✓	✓	✓				✓	
Albashabsheh and Stamm (2021)		✓			✓	✓		✓		
Abbasi et al. (2021)			✓		✓		✓	✓		
Mottaghi et al. (2022)	✓	✓	✓	✓	✓	✓		✓		
Makepa et al. (2023)		✓			✓		✓	✓		
This study	✓	✓	✓	✓	✓		✓		✓	✓

As observed, despite efforts to study BSCR, there is a lack of systematic review, classification, analysis, identification of research gaps, and suggestion of potential directions for future research specifically focused on uncertainties in all types of biofuel networks. In addition, few research papers deeply focused on uncertainty modelling and discussed the current limitations. To address these gaps, this paper aims to conduct a comprehensive review of 205 relevant papers, selected from an initial screening of 1730 papers published up to 2022, with a specific focus on the modelling of uncertainties and disruptions in BSCs. The study covers various aspects, including:

- Examination of all types of BSCs, ranging from first to fourth generations of biofuels.
- Intense focus on uncertainties, encompassing the sources of uncertainties and quantitative solutions to address them.
- Discussion of recent developments in the field and identification of potential directions and research gaps for future investigations.

### Study objectives

The primary emphasis and contribution of this research are centred on the research questions listed below:

- RQ1: How did the literature model the uncertainties in BSC problems?
- RQ2: What are the drawbacks of the existing quantitative approaches to model the uncertainties in BSC problems?
- RQ3: What are the missing aspects in the relevant literature that have the potential for future research?

To address these research questions, this study categorizes and examines existing theories and methodologies employed in BSC research to tackle uncertainties and disruptions (RQ1). The limitations of the current literature are acknowledged, along with suggestions on how they can be addressed (RQ2). Moreover, critical research gaps are identified, and potential future directions are proposed based on survey statistics (RQ3). This study contributes to a better understanding of the uncertain sources and suitable approaches to face them for decision-makers. It also provides new directions for future research and paves the way for researchers to come up with breakthrough ideas and address existing limitations in managing uncertainties in BSCs. Obviously, the results of this study will protect the health of BSCs, which significantly impacts sustainability in today’s world, and help societies avoid the harmful effects of fossil fuels through developing efficient BSC networks.

The subsequent sections of the paper are structured as follows. The “Review methodology” section discusses the methodology followed to create the shortlist of papers for literature review. The “Macro-level analysis and data visualization” section provides detailed information about the publication year, journals, and geographical origins of shortlisted papers. The “Supply chain structure and uncertain environment” section analyses and categorizes the shortlisted papers regarding supply chain structure and uncertain environment, uncertainty sources, and methodologies for facing uncertainties and disruptions. The “Discussion of current research gaps and future research directions” section lists the current research gaps and future research directions. Finally, the closing thoughts and outcomes are outlined in the “Conclusion” section.

## Review methodology

**Fig. 2:**
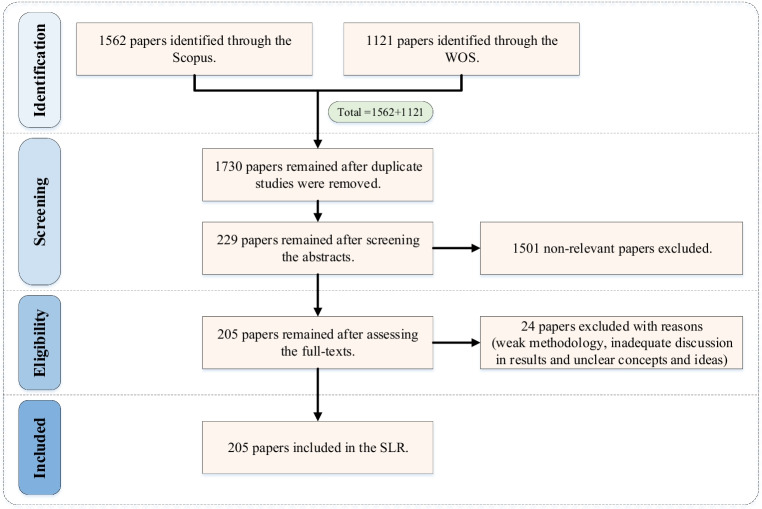
The PRISMA flowchart followed to identify the suitable papers for SLR

A comprehensive survey of literature in the field of BSCR is conducted to evaluate the existing body of research and consolidate the scholarly efforts in this domain. This systematic literature review (SLR) is implemented based on the Preferred Reporting Items for Systematic Reviews and Meta-Analyses (PRISMA) method (Martucci et al. 2023). This methodology involves several advantages, such as considering the inclusion and exclusion criteria and evaluating extensive literature databases in a predetermined period (Jamaluddin and Saibani 2021). Figure 2 represents different stages of PRISMA that were followed to select the articles needed for our SLR.

The research process is outlined in detail as follows:

- Step 1: defining the rules
  The review process covers the journal articles, including published, in-press, and pre-publication versions, written in English about BSC problems where quantitative methodologies to address uncertainties have been proposed. This means that conference papers are not included here due to their lack of accessibility and different review processes. There is no limit on the publication year of papers; all studies published by 2022 are covered.
- Step 2: selecting query and database

In this stage, we started by creating an initial set of keywords (e.g. resilien*, biofuel, and supply chain) to identify a few initial papers investigating BSCR. This procedure was carried out to select ten papers published in high-quality journals. Then, their keywords and derivatives were added to the initial list of keywords to find other relevant research items. These steps were continued to expand the keywords list until it became mature enough and included all high-frequent keywords. The final list of keywords is as follows:

- (supply chain* OR SC OR logistic* OR supply network* OR distribution network*)
- AND (resilien* OR disrupt* OR uncertain* OR risk* OR threat* OR failure* OR vulnerab* OR hazard* OR catastroph* OR disast* OR intrupt* OR crisis OR disturbance*)
- *AND (biofuel OR bio-fuel OR biomass OR bio-mass OR biodiesel OR bioethanol OR biogas)*

As observed, similar terms are fallen into the same category using the Boolean operator “OR” and Boolean operator “AND” puts these three categories together. In order to ensure impartiality and comprehensiveness, two online datasets, Scopus and Web of Science (WOS), were utilized to identify relevant papers. These two sources cover almost 95% of all research publications, providing a comprehensive information base (Spieske and Birkel 2021). It is worth mentioning that the query was used to search within the titles, abstracts, and/or keywords of articles.

- Step 3: selecting papers

The selected online databases were thoroughly searched using the specified search query and the conditions outlined in the previous steps. Based on the search, the number of records found was 1562 in Scopus and 1121 in WOS. Meanwhile, if the keywords related to biofuel (third category) were removed, these values would reach 356,812 and 247,224, respectively. This dramatic difference shows that a small percentage of papers that discussed supply chain uncertainty and disruption were devoted to the BSC. Then, out of 2683 articles found, 953 duplicates were identified and removed. The titles and abstracts of the remaining 1730 articles were screened, and 1501 items were discarded since they did not satisfy the predetermined inclusion conditions. Next, the eligibility evaluation was performed by attentively screening the full texts of the 229 remaining articles. Here, 24 papers were excluded for several reasons, including weak methodology, inadequate discussion of results, and lack of transparency in concepts and ideas. Finally, 205 records that clearly focused on uncertainties and disruptions in BSC, meeting our mentioned criteria, were selected for further analysis.

## Macro-level analysis and data visualization

The shortlisted papers are examined in terms of their publication period, journals, and geographical distribution. Further details regarding these aspects are discussed in the subsequent sections.

### Classification based on publication period

**Fig. 3:**
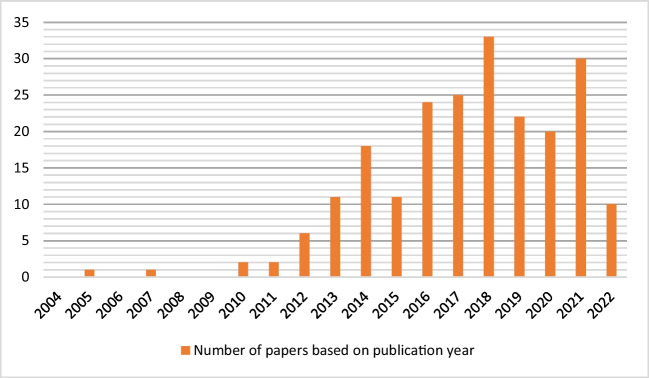
The publication years of shortlisted papers

The detailed information of the publication period can represent the scholarly attention received by the BSCR problem over time. Therefore, an analysis of the publication years of the shortlisted papers is presented, with the results illustrated in Fig. 3. According to this figure, the number of published papers in this field has had an upward trend, indicating the importance of this problem in recent years. Although a few papers were published from 2004 to 2009, the number of papers has increased since 2010 to reach its peak in 2018. The upward trend indicates the importance of this problem in recent years.

### Classification based on publication journal

The number and percentage of papers published in each journal are shown in Table 3 in the Appendix. The range of the journals that published these papers is almost wide and includes 72 journals. The *Journal of Cleaner Production*, *Computers and Chemical Engineering*, *Energy*, *Applied Energy*, *Biomass and Bioenergy*, *Chemical Engineering Transactions*, *Computers and Industrial Engineering*, and *Transportation Research Part E: Logistics and Transportation Review* published 23, 21, 18, 11, 9, 9, 7, and 6 papers that have the largest share, respectively, and covered a total of almost 50% of published papers.

### Classification based on the geographical distribution

**Fig. 4:**
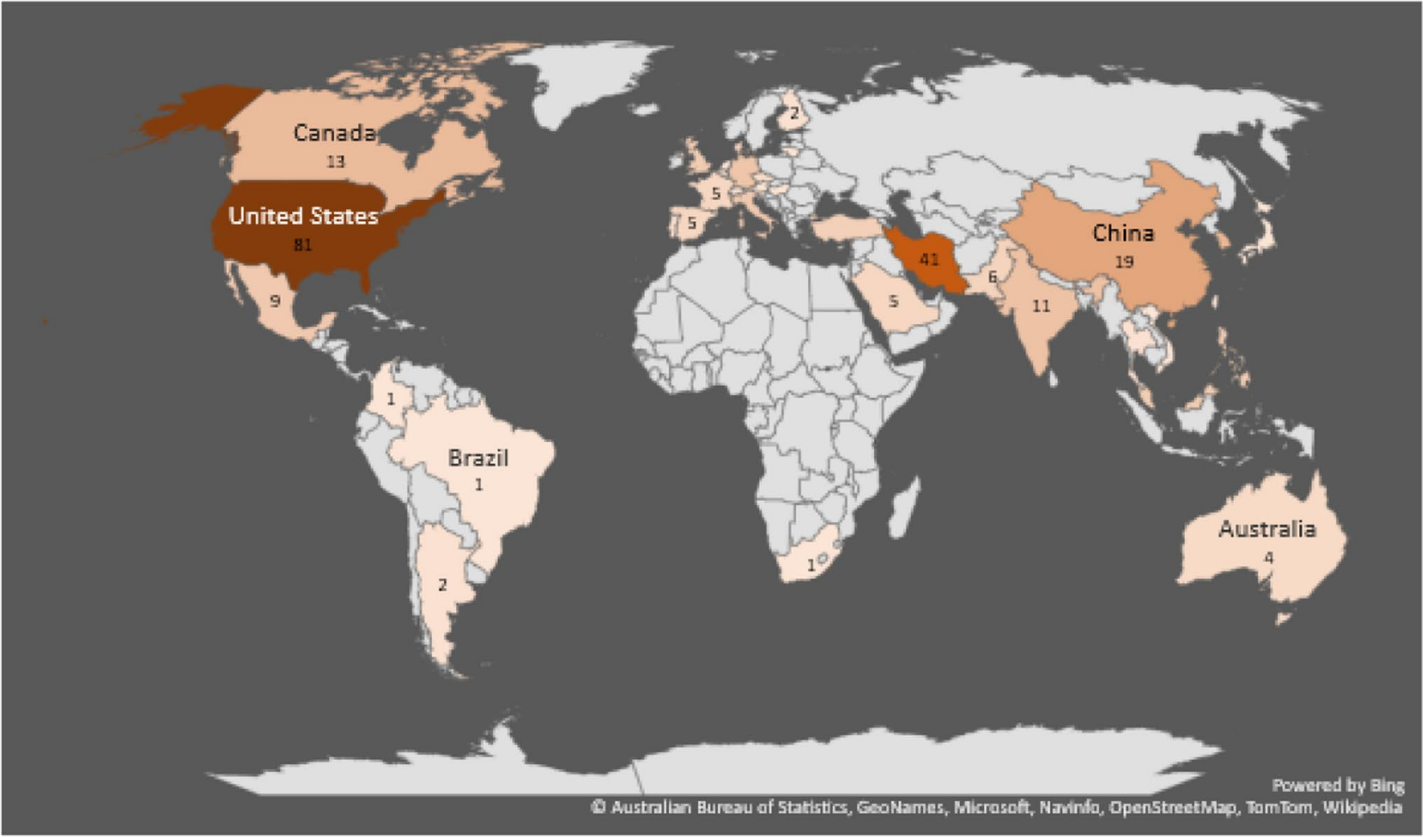
Geographical distribution of contributing countries

Based on the authors’ affiliations, Fig. 4 depicts the distribution of papers published across different geographical locations worldwide. The contribution degree of each country is reflected by colourfulness. Thirty-eight countries have been active in publishing scientific papers in this area. The research institutes in the USA played a prominent role in extending the body of knowledge in BSCR. In addition, our statistics indicated that Iran, China, South Korea, Canada, India, and Germany are the following productive countries that come next places, respectively.

## Micro-level analysis of the papers

This section analyses and categorizes the shortlisted papers according to three criteria: the supply chain structure and uncertain environment, sources of uncertainties, and methodologies to face uncertainties and disruptions.

### Supply chain structure and uncertain environment

As previously mentioned, feedstocks in a BSC network are processed into biofuels and distributed to end consumers. It consists of several sorts of facilities, each performing a distinct function in the network. A layer, tier, or echelon define as a group of facilities that perform the same operation and are of the same type (Vanbrabant et al. 2023). Biomass/feedstock production, pre-treatment facility, feedstock storage, biofuel conversion facility/biorefinery, and consumers are the main layers of a typical BSC network. Material flows commonly occur from biomass/feedstock production facilities to consumers. This type of material flow is known as the forward flow, which is widely used by papers (Arabi et al. 2019a; Hombach et al. 2016; Zerafati et al. 2022). On the other hand, the material flow might be from the downstream layers to the upper one, defined as the reverse or backward flow (Abasian et al. 2019; Mohseni and Pishvaee 2016). For example, the *Jatropha curcas* press cake, which includes almost 55% of the seeds, is transported from refineries to the fields for fertilization in the biodiesel supply chain model investigated by Awudu and Zhang (2013). In addition, several studies considered the material flow in a tier of the BSC network known as the intra-layer flow or lateral transshipment. Yang et al. (2020) investigated a three-layer coupled network encompassing electric and biogas distribution chains to address uncertainties in feedstock supply and energy demand while achieving a harmonious equilibrium. Their network included biomass supply nodes, substations, and demand nodes, where each layer is interconnected.

Some papers in BSCR also highlighted the considerable need for quick recovery after the disruptions so that the severe damage of ripple effect propagating throughout the supply chain network is reduced (Benjamin 2017, 2018; Salehi et al. 2022). The ripple or domino effect refers to the impacts of disruptions propagating on the efficiency of the supply chain, which mainly depends on supply chain structural design and planning parameters. In other words, the ripple effect happens when disruption cascades downstream and affects the efficiency of the BSC rather than being localized or confined to one section of the network. This influence may include delays in distribution and production, decreasing sales, and damage to the biofuel market. The share of shortlisted papers that examined the ripple effects in the BSC was 3.4%, much less than in other fields.

The primary purpose of the BSCR studies is to analyse the system to find a suitable design or improve (redesign) the current configuration so that the system’s elements perform well under uncertain conditions. However, this good performance relies on the uncertain environment, depth of uncertainties, and the methodology used to face those.

According to the availability level of information for decision-making purposes, the uncertain environments for BSCR problems can be classified into three categories as follows (Govindan et al. 2017; Nimmy et al. 2022):

- The condition where there is no data regarding the probabilities of uncertain parameters (C_1_): here, robust optimization techniques are often developed to optimize the worst-case functionality of the BSC network.
- The condition where the probabilities of uncertain parameters are available (C_2_): this type of uncertain parameter, referred to as stochastic, can be defined by continuous or discrete scenarios. The stochastic programming approach is one of the most common techniques to face uncertainties triggered by stochastic parameters.
- The condition where uncertain parameters are either vague or ambiguous (C_3_): two concepts of ambiguity and vagueness are defined for this category. Ambiguity is when a decision among several options is undetermined, but vagueness denotes the condition where crisp and precise borders for some areas are not determined. Fuzzy modelling can handle uncertainties in these two cases by defining membership functions.

Further discussion of these techniques will be provided in the “Mathematical modelling” section.

### Uncertain sources and parameters

Disruptions can occur and start from any part of the supply chain structure since uncertainties exist in all parts of the BSC network. Table 2 lists the primary uncertain parameters and sources of disruptions involved in planning the BSC networks in the studied papers. In this table, the uncertain parameters are classified into seven groups. The fourth column of this table indicates the reference papers where those uncertain parameters were studied. Table 2 also illustrates the frequency of studied uncertain parameters, which serves as a critical factor in understanding the scope of uncertainties. It should be noted that the total percentage may not add up to 100% as some studies consider multiple sources of uncertainty.

**Table 2:** Details of the uncertain parameter in shortlisted BSCR papers

Row	Category	Uncertain parameter	Reference(s)	Frequency (%)
1	Supply side	Feedstock supply/availability	(Abriyantoro et al. 2019; Aghalari et al. 2021; Ahmed and Sarkar 2018, 2019; Ahn and Kim 2021; Ahranjani et al. 2020; Alizadeh et al. 2019; Allman et al. 2021; Arabi et al. 2019b; Azadeh and Arani 2016; Babazadeh 2018, 2019; Balaman 2016; Balaman et al. 2018; Balaman and Selim 2014b, 2015, 2016; Bär et al. 2017; Benjamin 2018; Carvajal et al. 2019; Chen et al. 2022; Chen and Fan 2012; Díaz-Trujillo et al. 2020; Ebadian et al. 2014; Espinoza-Vázquez et al. 2021; Fan et al. 2019; Fattahi and Govindan 2018; Fattahi et al. 2021; Foo et al. 2013; Gao and You 2017a, b; Garai et al. 2021; Gebreslassie et al. 2012; Geismar et al. 2021; Geng et al. 2021; Ghadge et al. 2020; Ghelichi et al. 2018; Giarola et al. 2012; Gonela^2018^; Gonela et al. 2015a; b; Gumte et al. 2021; Guo et al. 2022; Habib et al. 2021, 2022; Hombach et al. 2018; Hong et al. 2014; Hu et al. 2017; Huang et al. 2014; Jana et al. 2022; Kazemzadeh and Hu 2013; Khezerlou et al. 2021; Khishtandar 2019; Kim et al. 2011; Lambert et al. 2021; Li and Hu 2014; Li et al. 2017; Liu et al. 2017; Lo et al. 2020, 2021; Maheshwari et al. 2017; Mamun et al. 2020; Martinkus et al. 2017; Marufuzzaman and Ekşioğlu 2017; Marufuzzaman et al. 2014a; Memişoğlu and Üster 2021; Mirhashemi et al. 2018; Mirkouei et al. 2017; Mohammadi et al. 2023; Mousavi Ahranjani et al. 2018; Ngan et al. 2019, 2020; Nguyen and Chen 2018, 2022; Osmani and Zhang 2013, 2014a, 2017; Paulo et al. 2015; Pavlou et al. 2016; Poudel et al. 2016b, 2019; Quddus et al. 2017, 2018; Razm et al. 2021; Ren et al. 2015, 2016; Reyes-Barquet et al. 2022; Rezaei et al. 2020; Rungphanich and Siemanond 2019; Saghaei et al. 2020a, b; Sahoo et al. 2018; Sajid 2021; Santibañez-Aguilar et al. 2018; Senna et al. 2016; Shabani and Sowlati 2016a; Shabani et al. 2014; Sharifzadeh et al. 2015; Sharma et al. 2013, 2020; Shavazipour et al. 2020; Soren and Shastri 2019, 2021; Tan et al. 2016; Tong et al. 2014a, b, c; Üster and Memişoğlu 2018; Yang et al. 2020; Ye et al. 2017;2018; Yue and You 2016a, b; Zamar et al. 2017; Zarei et al. 2022; Zhang et al. 2017a, b; Zhang et al. 2016; Zirngast et al. 2019)	58.05%
2		Feedstock quality	(Abriyantoro et al. 2019; Aghalari et al. 2021; Castillo-Villar et al. 2017; Karimi et al. 2021; Lo et al. 2021; Mamun et al. 2020; Nur et al. 2021; Reyes-Barquet et al. 2022; Saghaei et al. 2020a, b; Shabani and Sowlati 2016b; Zirngast et al. 2019)	5.85%
3		Feedstock demand	(Khishtandar 2019; Ngan et al. 2020; Shabani and Sowlati 2016a)	1.46%
4		Import price	(Gumte et al. 2021)	0.49%
5		Harvest rate	(Ghaderi et al. 2018)	0.49%
6	Processing	Conversion rate/efficiency	(Bairamzadeh et al. 2018; d'Amore and Bezzo 2017; Garai et al. 2021; Gonela 2018; Hombach et al. 2018; Mirhashemi et al. 2018; Tong et al. 2014a; Xie and Huang 2013; Yue and You 2016a, b)	4.88%
7		Technical factors	(Mohseni et al. 2016)	0.49%
8		Production factors (expect cost)	(Biwer et al. 2005; De Meyer et al. 2015; Geng et al. 2015; Razm et al. 2021)	1.95%
9		Production/processing/operational capacity	(Azadeh and Arani 2016; Bär et al. 2017; Benjamin et al. 2017, 2021; Fallah and Nozari 2021; Fattahi and Govindan 2018; Hombach et al. 2018; Li et al. 2017; Pavlou et al. 2016; Pinho et al. 2021; Salm et al. 2017; Sharma et al. 2018; Zhao and You 2019)	6.34%
10		Refinery/conversion operations	(Li et al. 2017; Hu et al. 2017; Huang and Pang 2014; Khezerlou et al. 2021; López-Díaz et al. 2018)	2.44%
11	Costs	Transportation cost	(Balaman 2016; Balaman et al. 2018; Balaman and Selim 2014b; Fallah and Nozari 2021; Habib et al. 2021; Hu et al. 2017; Kanan et al. 2022a; Kazemzadeh and Hu 2013; Kim et al. 2011; Lo et al. 2020, 2021; Mohseni and Pishvaee 2016; Paulo et al. 2015)	6.34%
12		Feedstock purchase cost/price	(Balaman et al. 2018; Biwer et al. 2005; Habib et al. 2020; Hombach et al. 2018; Khishtandar 2019; Lee et al. 2017; Lo et al. 2020, 2021; López-Díaz et al. 2018; Ngan et al. 2020; Ning et al. 2018; Ning and You 2019; Osmani and Zhang 2013, 2014a; Santibañez-Aguilar et al. 2015, 2016b; Shabani and Sowlati 2016a; Ye et al. 2018; Zhang et al. 2017a, b; Zirngast et al. 2019)	10.24%
13		Production/operational cost	(Ascenso et al. 2018; Balaman et al. 2018; Balaman and Selim 2015; Dal-Mas et al. 2011; Hombach et al. 2018; Kim et al. 2011; Mirhashemi et al. 2018; Mohseni and Pishvaee 2016; Paulo et al. 2015; Tong et al. 2014a)	4.88%
14		Capital/establishment cost	(Balaman 2016; Balaman et al. 2018; Balaman and Selim 2014b, 2015; Kim et al. 2011; Mirhashemi et al. 2018; Ngan et al. 2019; Paulo et al. 2015)	3.90%
15		Inventory cost	(Habib et al. 2021; Mirhashemi et al. 2018)	0.98%
16		All costs	(Babazadeh 2018; Babazadeh et al. 2017; Balaman and Selim 2014a, 2016; Ghaderi et al. 2018; Habib et al. 2022; Mohseni et al. 2016; Mousavi Ahranjani et al. 2018; Ngan et al. 2019; Razm et al. 2021; Rezaei et al. 2020; Sharifi et al. 2020)	5.85%
17		Recycling cost	(Geng et al. 2018)	0.49%
18	Demand side	Biofuel/final product price	(Abasian et al. 2019; Awudu and Zhang 2013; Azadeh and Arani 2016; Azadeh et al. 2014; Bairamzadeh et al. 2016; Balaman et al. 2018; Balaman and Selim 2014b; Biwer et al. 2005; Dal-Mas et al. 2011; Dal Mas et al. 2010; Diehlmann et al. 2019; Gebreslassie et al. 2012; Gilani and Sahebi 2021; Gonela et al. 2015a, b; Hasanly et al. 2018; Höltinger et al. 2014; Hombach et al. 2018; Jana et al. 2022; Kalhor et al. 2023; Kazemzadeh and Hu 2013; Kim et al. 2011; Kostin et al. 2010; Li and Hu 2014; Lo et al. 2020, 2021; López-Díaz et al. 2018; Marvin et al. 2012; Mousavi Ahranjani et al. 2018; Ng et al. 2013; Ngan et al. 2020; Osmani and Zhang 2013, 2014a, b, 2017; Santibañez-Aguilar et al. 2016a; Shabani and Sowlati 2016a; Shavazipour et al. 2020; Tong et al. 2014b; Yeh et al. 2015; Zhang and Jiang 2017; Zhang et al. 2016)	20.49%
19		Final product demand	(Abasian et al. 2019; Ahmed and Sarkar 2018; Ahn and Kim 2021; Asadi et al. 2018; Awudu and Zhang 2013; Azadeh and Arani 2016; Babazadeh 2018; Babazadeh et al. 2019; Bairamzadeh et al. 2016; Benjamin 2017, 2018; Chen and Fan 2012; De Meyer et al. 2015; Delkhosh and Sadjadi 2020; Díaz-Trujillo et al. 2020; Diehlmann et al. 2019; Duc et al. 2021; Espinoza-Vázquez et al. 2021; Fallah and Nozari 2021; Fan et al. 2019; Gao and You 2017a, b; Geng et al. 2018; Ghaderi et al. 2018; Ghelichi et al. 2018; Gilani and Sahebi 2021; Gonela et al. 2015a, b; Gumte et al. 2021; Habib et al. 2020, 2021, 2022; Habibi et al. 2018; Hombach et al. 2018; Hwangbo et al. 2018a, b; Jana et al. 2022; Kalhor et al. 2023; Kanan et al. 2022a, b; Khezerlou et al. 2021; Kim et al. 2011; Kostin et al. 2010, 2012; Li et al. 2017; Lo et al. 2020; López-Díaz et al. 2018; Mirhashemi et al. 2018; Mobini et al. 2013; Mohseni and Pishvaee 2020; Mohseni et al. 2016; Mousavi Ahranjani et al. 2018; Ngan et al. 2020; Nguyen and Chen 2022; Ning et al. 2018; Osmani and Zhang 2013, 2014a, 2017; Razm et al. 2019,2021; Ren et al. 2015, 2016; Rezaei et al. 2020; Rungphanich and Siemanond 2019; Saghaei et al. 2020a, b; Salehi et al. 2022; Savoji et al. 2022; Sharifi et al. 2020; Sharifzadeh et al. 2015; Shavazipour et al. 2020; Tong et al. 2014a, b, c; Xie and Huang 2018; Yang et al. 2020; Ye et al. 2018; Yeh et al. 2015; Yue and You 2016a, b; Zamar et al. 2017; Zarei et al. 2022; Zhang et al. 2017a, b; Zhao and You 2019)	41.46%
20		Alternative fuel price	(Arabi et al. 2019a; Tong et al. 2014b)	0.98%
21	Resources	Required resource price	(Ahmed and Sarkar 2018; Balaman and Selim 2014b; Biwer et al. 2005)	1.46%
22		Resource supply/availability	(Balaman and Selim 2014a; Hombach et al. 2018; Mohseni and Pishvaee 2016; Mohseni et al. 2016; Mota-López et al. 2019; Ngan et al. 2019; Tan et al. 2016)	3.41%
23	Environmental and social	Emission	(Ascenso et al. 2018; Balaman et al. 2018; Habibi et al. 2021; Hombach et al. 2018; Savoji et al. 2022)	2.44%
24		Environmental impact	(Babazadeh et al. 2017; Bairamzadeh et al. 2016; Ghaderi et al. 2018; Habibi et al. 2018; Mousavi Ahranjani et al. 2018; Ngan et al. 2019; Rezaei et al. 2020)	3.41%
25		Carbon trading/market	(Giarola et al. 2012, 2013)	0.98%
26		Carbon tax rate	(Alizadeh et al. 2019)	0.49%
27		Social impact	(Ghaderi et al. 2018; Habibi et al. 2018; Mousavi Ahranjani et al. 2018; Ngan et al. 2019)	1.95%
28		Economic impact	(Habibi et al. 2018)	0.49%
29	Others	Restrictions/regulations/policies	(Diehlmann et al. 2019; Garai et al. 2021; Hombach et al. 2016, 2018; Ngan et al. 2019)	2.44%
30		Overall performance of facilities	(Liu et al. 2017; Marufuzzaman et al. 2014a; Poudel et al. 2018; Tan et al. 2016)	1.95%
31		Connection between facilities/transportation	(Gilani et al. 2020; Khezerlou et al. 2021; Marufuzzaman and Ekşioğlu 2017; Poudel et al. 2016a; Salimi and Vahdani 2018; Sarkar et al. 2021)	2.93%
32		Income/profit	(Babazadeh 2018; Balaman and Selim 2014a, 2016; Ngan et al. 2019)	1.95%
33		Delivery/transportation time	(Abriyantoro et al. 2019; Asadi et al. 2018; Balaman et al. 2018; Fallah and Nozari 2021; Habibi et al. 2018)	2.44%
34		Transport distance	(De Meyer et al. 2015)	0.49%
35		Exchange rate/currency fluctuations	(Razm et al. 2019; Shavazipour et al. 2020)	0.98%
36		Return percentage	(Garai et al. 2021)	0.49%
37		Technology advancements/developments	(Diehlmann et al. 2019; Li and Hu 2014; Marufuzzaman et al. 2014a; Ngan et al. 2019; Tong et al. 2014b; Walther et al. 2012)	2.93%
38		Losses due to transportation	(Hu et al. 2017)	0.49%
39		Transportation capacity	(Jana et al. 2022; Ren et al. 2016)	0.98%
40		System/market structure	(Burli et al. 2021)	0.49%
41		Available workforce	(Khishtandar 2019)	0.49%

### Methodologies to face uncertainties and disruptions

This subsection examines various techniques employed in addressing the inherent uncertainties in the parameters, as outlined in the shortlisted papers. Based on their capabilities, they are categorized into five main groups, including mathematical modelling, simulation, network-based approaches, machine learning, and other techniques. Although they are different in handling uncertainties, their similarity is that they all try to obtain suitable decisions by considering the effect of uncertainties.

#### Mathematical modelling

Mathematical modelling is a critical component of decision-making assistance in various policy processes, particularly those focused on uncertainty and disruption management (Almeida et al. 2018; Makowski 2005). Different mathematical models can be designed to optimize the BSC network under uncertain conditions according to the uncertain environment. The primary distinction between these approaches stems from the various concepts developed to represent the uncertainty of input data (Soroudi and Amraee 2013). For instance, the result of the stochastic programming technique is based on the probability distribution functions considered for parameters, while fuzzy approach models describe these uncertainties as membership functions. While the techniques discussed in the following section differ from one another, they share a common goal of quantifying the impact of input parameters on outputs. The ensuing discussion delves into these techniques in detail.

##### Stochastic programming

Stochastic programming is the most frequent method in BSCR, which represents and considers any feature prone to uncertainty, fluctuation, and risk using probability distribution functions of stochastic parameters. This approach aims to optimize the system by identifying optimal decisions that either minimize or maximize one or more objective functions. Typically, the most frequently employed objective function in this context is minimizing costs or maximizing profits.

This methodology assumes that the probability of the distribution function is available (Geismar et al. 2021; Lo et al. 2023) or at least estimable in advance (Díaz-Trujillo et al. 2020; Osmani and Zhang 2014b), which can be continuous (Castillo-Villar et al. 2017; Ye et al. 2017) or scenario-based (Gonela et al. 2015a; Zarei et al. 2022). When the problem is dealing with a continuous distribution function, only one uncertain parameter is usually involved, and the estimation and analysis are carried out according to the datasets and historical data. In the case of BSCR, this parameter is usually either biofuel demand or feedstock supply. Since considering the continuous distribution function usually results in the complexity of the solving problem, the values may be considered finite and discrete, where each value is known as a scenario with its probability. Considering M/M/1 queuing system to address demand and feedstock seasonality uncertainties, Khezerlou et al. (2021) designed a resilient BSC network where facilities, including a multimodal terminal and biorefineries, and their links are prone to disruption risk. They used variable transitional probabilities and conditional value at risk (CVaR) to model reliability and resiliency in their problem. However, their approach needs failure probabilities in all nodes (facilities) and arcs (transportations) that might be difficult to achieve in real-work conditions.

Two-stage stochastic optimization is the most popular methodology in BSCR and falls within the stochastic programming category. This methodology makes stage-one decisions (usually long-term ones) in advance, resulting in evaluating probable outcomes; therefore, stage-two or corrective actions (usually short-term decisions) are taken at the end of the period (Li and Grossmann 2021). Giarola et al. (2013) proposed a two-stage stochastic model where capital investment and technology selection decisions were determined in the “here-and-now” mode. Then, the operational decision, such as procurement and sales, was made in the “wait-and-see” stage. While this technique yields reliable results by making second-stage decisions after clarifying the situation, the computational requirements significantly increase with the growing number of uncertain parameters (Mavromatidis et al. 2018). In some cases, this approach involves more than two stages, referred to as multistage stochastic programming. Fattahi and Govindan (2018) presented a multistage stochastic programming model for a four-layer BSC network where feedstock availability is considered a random parameter. They solved their model for a timeframe of (*t*, *t* + *1*, *t* + *2x*,* …*,* T*) at the start of the planning horizon (*t*) to determine the optimal policy for period *t*. After realizing uncertainties in period *t*, the model will be run for the rest periods. This procedure continued until the final results were obtained for the last period (T). The main disadvantage of this technique is the complex calculations when the number of uncertain parameters increases (Mavromatidis et al. 2018).

Chance-constrained programming is another stochastic-based optimization technique that aims to meet soft constraints with a predefined probability, known as the reliability level (Baryannis et al. 2019). Lambert et al. (2021) used this method to meet the biorefinery capacity constraint when it can be affected by uncertainty in feedstock flow. Although chance-constrained programming can efficiently handle uncertainties thanks to its robustness, the probability distribution functions are sometimes hard to formulate, particularly in non-linear problems.

##### Robust optimization

The robust optimization is an appropriate approach to face uncertainties when the probability distribution function for uncertain parameters is not available or estimable. Robust techniques empower the BSC network to resist operational variations, preserve its structure, remain efficient, and guarantee consistent performance (Behzadi et al. 2017). The primary objective of robust optimization is to generate a reliable result and solution by considering the worst-case scenario. Since the worst-case situation is considered while optimizing the network, the results of robust optimization will be less vulnerable or even immune to variations under uncertain conditions (Kalhor et al. 2023). Robust optimization methodologies may be separated into two categories depending on how the uncertainty of input data is represented:

- Scenario-based methods: these approaches incorporate uncertainties using a set of discrete scenarios. This approach offers the advantage of effectively managing the level of robustness and ensuring feasibility (Salimian and Mousavi 2022). However, it can be quite challenging when many scenarios are available due to a lack of information and data (Ahmadvand and Sowlati 2022). Delkhosh and Sadjadi (2020) introduced a two-phase optimization framework for third-generation biofuel supply chains (BSCs). In the macro-phase, the best cultivation system was selected using the best–worst method (BWM). Then, the economic and environmental performance of the network was optimized in the micro-stage using a scenario-based robust technique where demand was considered the uncertain parameter.
- Interval-based techniques: these techniques consider that potential values of uncertain parameters are contained inside a continuous uncertainty set. For instance, Mohseni and Pishvaee (2016) considered the uncertainties in operational and transportation costs as intervals due to the nature of these parameters.

There are two criteria used to assess the results of robust optimization techniques, including model robustness and solution robustness. The former measures the solution’s feasibility to examine which constraints are unsatisfied. However, the latter measures the solution’s closeness to the optimal one by evaluating the objective function (Baryannis et al. 2019).

##### Fuzzy modelling

Risk management encompasses three types of uncertainties: deep, random, and epistemic uncertainties. Deep uncertainty arises when the probability distribution of a parameter cannot be estimated due to limited data and information, although the boundaries may still be estimable. Random uncertainty pertains to the inherent irreducible randomness of parameters, and its probability distribution can be estimated using historical data. On the other hand, epistemic uncertainty is reducible and stems from insufficient or flawed data, measurement limitations, and estimations (Ghaderi et al. 2018; Mohammadi et al. 2023). Fuzzy modelling is a practical tool to face epistemic uncertainties where there is a lack of information about the precise value of parameters like feedstock availability (Mohammadi et al. 2023), bioproduct prices (Balaman et al. 2018), conversion rate (Tong et al. 2014a), GHG emissions (Balaman et al. 2018), costs (Babazadeh et al. 2017), amount of harvested algae (Arabi et al. 2019b), and biofuel demand (Ahmed and Sarkar 2018; Fallah and Nozari 2021). When human perceptions and opinions are used in the decision-making procedure, systematic uncertainty emerges owing to a lack of comprehensive understanding of the critical parameters, constraints, and goals (Naderi et al. 2016). These systematic uncertainties can be described using membership functions. As mentioned by Ghaderi et al. (2018), two categories of fuzzy mathematical modelling can be used separately or together in supply chain problems:

- Possibilistic programming: this approach is utilized when there is no access to the information and historical data regarding the actual values of parameters, but they can be explained using possibilistic distributions. For example, Tong et al. (2014a) developed a fuzzy possibilistic programming approach to design a hydrocarbon BSC where possibility, necessity, and credibility criteria are modelled according to the decision-makers’ desires. A key drawback of this approach is its formulation solely based on average-case conditions, where decisions are made according to expected values while disregarding risk values (Babazadeh 2019).
- Flexible programming: this technique manages fuzzy constraints and goals resulting from the decision maker’s imprecise preferences. This implies that a decision-maker may prioritize flexibility, allowing for violations of soft constraints within certain limits, and adopt a flexible target for the objective function rather than strictly optimizing it. (Abusaq et al. 2022). Investigating such techniques in BSCR literature is quite rare and worth studying in future research.

##### Hybrid techniques of mathematical modelling

In an effort to integrate at least two of the previously mentioned methodologies into a unified framework, a small fraction of BSCR problems may not fit precisely into the subsections mentioned above. Therefore, we defined them as hybrid techniques since they involved at least two combinatorial methods. The first class, which has accounted for a significant percentage, used fuzzy sets in conjunction with robust (Ghaderi et al. 2018; Habib et al. 2021, 2020; Mousavi Ahranjani et al. 2018; Savoji et al. 2022) and stochastic optimization techniques (Alizadeh et al. 2019). Such techniques have the ability to model the problem when there are several parameters with different uncertain natures (i.e. deep, random, and epistemic uncertainties). As its strong point, Gilani et al. (2020) believed that fuzzy programming considering membership degrees have higher adaptability to model uncertainties of biofuel price and demand. Hence, they employed robust possibilistic programming to optimize a sugarcane-to-biofuel supply chain with sustainable considerations. Although they considered the uncertainties both in objective function and constraints simultaneously for the first time, the number of uncertain parameters involved in their problem was limited, and their focus was mainly on road access disruption.

The hybridization of fuzzy modelling and chance-constrained technique empowered Khishtandar (2019) to consider various uncertainties, including feedstock demand, availability, price, and manpower availability, in their problem to design a biogas supply chain network. However, they overlooked the seasonal variations in biomass transport from supply sources to hubs and subsequently to the reactor. As another suggestion, the disruption in facilities could make their problem more realistic while considering different uncertainties. Ahranjani et al. (2020) modelled the operational and disruption risks simultaneously in the BSC using the unique combination of fuzzy, robust, and stochastic approaches. Although they studied disruption risks alongside the epistemic uncertainties, other types of uncertainties (i.e. deep and random uncertainties) could be involved in their problem. The same approach was followed by Sharifi et al. (2020) to face uncertainties in costs and biofuel demand in the second-generation BSC. The recent research trend underscores the significance of employing hybrid techniques to address uncertainties from multiple sources, thereby aligning the problem with real-world scenarios. Other types of hybridization exist in the literature devoted to the combination of multi-criteria decision-making (MCDM) and robust optimization (Razm et al. 2021), MCDM and stochastic programming (Mirkouei et al. 2017), stochastic programming and Monte Carlo experiment (Zirngast et al. 2019), and stochastic and robust optimization (Kalhor et al. 2022; Mirhashemi et al. 2018; Shabani and Sowlati 2016b; Yue and You 2016a, 2016b).

##### Other approaches to mathematical modelling

Several distinctive mathematical modelling methodologies have been proposed within the field of BSCR. Most papers in this class studied a pre-disaster planning mathematical model to design a reliable BSC when a failure probability exists. However, they cannot be categorized into the previously mentioned methodologies (Bai et al. 2015; Liu et al. 2017; Maheshwari et al. 2017; Marufuzzaman and Ekşioğlu 2017; Marufuzzaman et al. 2014b; Poudel et al. 2016a; Salimi and Vahdani 2018; Soren and Shastri 2019, 2021). The second category is those papers that design the model considering the complete information available for all parameters, and they only performed a sensitivity analysis to observe the effect of changes in parameters (Ascenso et al. 2018; Ge et al. 2021; Geng et al. 2018; Li et al. 2017; Mirhashemi et al. 2018; Zhang et al. 2022). The others developed unique methodologies to face uncertainties in the BSC network, such as regret theory by d’Amore and Bezzo (2017), game theory by Ye et al. (2017) and Zhang et al. (2017b), using GIS for optimization by Hu et al. (2017), two-stage adaptive robust fractional programming model by Zhao and You (2019), and Lagrangian relaxation by Nguyen and Chen (2022).

#### Simulation

The BSC essentially involves multiple interacting parties, each with distinct and potentially conflicting needs and objectives. Simulation serves as a suitable quantitative approach to analyse such environments, enabling the examination of system behaviour and the enhancement of relationships among entities. Simulation techniques present “what if” possibilities frequently used to study the system’s performance across time (Katsaliaki et al. 2022). They offer a flexible environment for modelling variability and recovery strategies, allowing for the incorporation of a high degree of complexity in the problem. Decision-makers may employ simulation to observe how the system reacts to various inputs, while optimization models only give clear advice in a specific situation. Simulation techniques used for BSCR problems are categorized into several groups.

The first and most common is the Monte Carlo simulation, which determines the responsivity of a system’s output by modelling the input parameters based on their probability distribution. This type of simulation uses iterative approaches and computations to obtain enough data for the related analysis (Benjamin et al. 2017). Lee et al. (2017) estimated the multivariable stochastic volatilities (SVs) and determined the common factors affecting the price of oil and agricultural products utilized for biofuel and other applications using the Markov Chain Monte Carlo technique. Benjamin et al. (2017) assessed the resilience of bioenergy parks when production capacity (or level) is prone to disruption. They used the Monte Carlo simulation approach to model the variation in the extent of disruption for each scenario. Lo et al. (2021) conducted a techno-economic feasibility analysis using the Monte Carlo simulation to assess the feedstock gasification procedure. The analysis considered various uncertainties, such as feedstock supply, price and quality, transportation cost, and sale price. Hasanly et al. (2018) used the Monte Carlo simulation to quantify the estimation risks at various biofuel prices and facility sizes for their designed bioethanol production system from wheat straw.

Lo et al. (2020) presented a Monte Carlo simulation approach to create a probability curve representing the uncertainties in transportation fuel price, feedstock price and availability, bioethanol price, and demand for technical and economical analysis of producing bioethanol from palm biomass. The use of the Monte Carlo simulation by Biwer et al. (2005) resulted in a detailed understanding of the effect of uncertainties on technical and network parameters in the biomass and penicillin V supply chain. A hybrid approach, including the MINLP model and Monte Carlo simulation, was proposed by Shabani and Sowlati (2016a) to optimize a forest-based supply network and assess the influence of feedstock quality, availability, price, and electricity costs on the system. Similarly, Benjamin et al. (2021) used this type of simulation to analyse the risk and evaluate the reliability of an integrated bioenergy system when processing capacity is prone to the risk of disruption. Mamun et al. (2020) used the Monte Carlo simulation to show that geographically distributed depots can efficiently absorb the risks due to uncertainties in a cellulosic BSC network. Santibañez-Aguilar et al. (2015) considered the uncertainty in raw material prices involved in the BSC. They generated stochastic scenarios utilizing the Latin hypercube approach alongside the Monte Carlo simulation to determine the suitable configuration for each sample scenario.

The second simulation approach utilized for BSCR management is system dynamics which, as a quasi-continuous modelling technique, investigates the complex, large, interdependent, and non-linear networks (Khanmohammadi et al. 2018). System dynamics as a technique provides valuable tools for analysing the dynamics and performance of supply chain networks. These tools include causal loop diagrams, which depict cause-and-effect relationships within the system. Mota-López et al. (2019) investigated the impact of water supply interruptions in a bioethanol supply chain network. They used system dynamics to analyse the system behaviour and demand satisfaction in four different time horizons. Using the same methodology, Ghadge et al. (2020) studied the effects of GHG concentration trajectories on bioethanol supply chains by defining eight scenarios for a 40-year time period. Salm et al. (2017) believed that the qualitative essence of the outcome in hazards and operability analysis considers a significant drawback. They overcame this barrier by assisting this analysis with a dynamic simulation approach to quantify and model the deviations and failures in a standard biogas production system.

Discrete-event simulation is the third category of simulation techniques used in this field, where only particular periods and conditions are applied to the object states and events (Paulo et al. 2022). The effect of operation disruptions, including machinery breakdowns, on BSC network performance, was assessed by B. Sharma et al. (2018) by proposing a database-centric discrete-event simulation. The combination of bale delivery and pellet delivery with biorefinery and depot uptime between 20 and 85% consisted of the scenarios they considered for analysis in a 7-year time period. In another related research, Mobini et al. (2013) estimated the time, cost, emission, and consumed energy in a wood pellet supply chain consisting of various entities and considered their interactions using discrete-event simulation modelling. Pavlou et al. (2016) developed three discrete-event simulation models to assess various feedstock harvesting, processing, and transship plans based on different machinery setups. Pinho et al. (2021) presented an event-based predictive model based on discrete-event simulation for coordinating long-term and short-term planning decisions in a forest-based BSC network.

Furthermore, another more novel simulation approach, agent-based modelling, recently attracted the researchers’ attention, and BSCR management was no exception. Agent-based modelling demonstrates a notable capability to analyse collaboration and dependencies among firms and participants, particularly in scenarios where multiple sources of uncertainties exist throughout the supply chain. Hence, it is highly recommended to be employed in future research in this area as it has received less attention compared to similar methods. Burli et al. (2021) designed an agent-based model to replicate farmer biomass crop adoption behaviour throughout a 50-county study area in Colorado. They examined the factors influencing farmers’ adoption choices, including individual and farm characteristics, market conditions, media influence, and social networks.

#### Network-based techniques

Because of the dynamic and complicated nature of the BSCR problem, particularly when faced with numerous uncertainties, some studies have adopted network-based models to describe the different potential states, their consequences, and probable transitions and relations among them. Firstly, Friedler et al. (1992) introduced P-graph, a graph-theoretical, combinatorial, and algorithm-based approach utilized to solve process network synthesis (PNS) problems. This technique offers several notable advantages, such as efficient data processing and output presentation through a graphical interface. Additionally, it has the capability to generate optimal and near-optimal solutions simultaneously (Sahl et al. 2023). Consequently, P-graph has lately extended into various research fields, and developing biomass supply chains under uncertain conditions has been no exception. Benjamin (2017) presented a developed methodology based on P-graph to analyse the uncertainties in demand for bioenergy parks. As an extension of the previous work, Benjamin (2018) used a technique based on P-graph to analyse the bioenergy parks when several disruptions occur in the supply and demand parts of the network. Their technique calculated the decline in net production caused by concurrent climate change-related events and market demand changes. A similar approach is presented by Tan et al. (2016) to identify suitable reactions to disruptions to minimize operational losses. The results show that the P-graph has an excellent ability for systematically planning operations against uncertainties and disruptions, especially for high combinatorial complexity problems where other quantitative approaches might have limitations (Ji et al. 2023). For example, employing the P-graph to generate suitable initial solutions for non-linear mathematical models can be a good choice for the investigation to increase decision-making efficiency.

The Bayesian network is another network-based technique used in BSCR problems. This methodology is a graphical model of probabilities which utilizes a directed acyclic network to describe a group of random variables (nodes) and their conditional connections (arcs) (Surendran et al. 2022). Bayesian networks cannot take temporal information into account, and they cannot simulate several phenomena throughout time. As a result, Dynamic Bayesian Network was proposed to address this limitation. Sajid (2021) investigated the effects of COVID-19 on the efficiency of the BSC network and feedstock availability to produce biofuel over 10 years. Bär et al. (2017) examined the potential of producing biofuels from woody and non-woody feedstocks in Tanzania. They used spatial Bayesian network modelling to consider uncertainties related to data and parameters. Despite the Bayesian network’s efficiency, there are a couple of disadvantages, including the expensive computations in the structure learning process and the inability to model cyclic relationships where generated data have at least three correlated variables (Hui et al. 2022). These limitations can be investigated for future research when employing the Bayesian network for BSC problems.

Furthermore, other network-based approaches have been employed in modelling the BSCR problems. Sahoo et al. (2018) assessed the availability of agricultural feedstocks at high geographical and time scales using the prediction models developed based on the Artificial Neural Network (ANN). The performance of ANN highly depends on the model’s parameter, where the suitable values of the weights lead to more accurate results. Utilizing metaheuristics to determine the suitable values can address this limitation. As another approach, Ngan et al. (2019) employed the Analytic Network Process (ANP) technique to analyse, assess, and rank the hazards commonly associated with the oil palm biomass production system.

#### Machine learning approaches

Machine learning (ML) algorithms may be used to automatize the resilience decisions and convert the conventional system of BSCR management into a dynamic process where prediction and learning play critical roles. However, the other mentioned techniques, such as mathematical modelling, lack the ability to learn and predict. ML methodologies have been exploited for several purposes in BSCR problems. The first research work devotes to S. Zhao and You (2020), where a new data-driven optimization approach based on deep learning was proposed. They used Generative Adversarial Network (GAN) to obtain the required distributional statistics (i.e. biofuel demand) from the available data, unsupervised and non-parametric. Then, the estimations were used in a robust chance-constrained programming model to design the overall structure of the BSC network. GAN’s primary disadvantage is the difficulties in training the model since various data types are required continuously to ensure the model acts accurately. Ning et al. (2018) combined machine learning and robust optimization by presenting a data-driven planning approach for the biofuel production system. They utilized Principal Component Analysis (PCA) to uncover underlying sources of uncertainty beyond the apparent ones and estimated their probability distributions using a kernel density estimation technique. Subsequently, these estimations were integrated into an adaptive robust model to optimize the bioproduct production system, taking into account uncertainties in bioproduct demand and feedstock price. PCA method will be biased when there are significant outliers (Bian et al. 2022). To address this limitation, it is suggested that they be eliminated from datasets before implementing PCA. Geng et al. (2015) modified the basic grey Markov model using the fuzzy approach to increase the model’s accuracy in predicting biofuel production. Chen et al. (2022) evaluated the efficiency of the ML predictive models and presented two ensembles (i.e. combinations of basic models), including linear and non-linear algorithms, to predict sugar yields. After screening through various regression measures, their proposed models consisted of the most suitable primary learners. While their model exhibits unique novelty, it may not be suitable for predicting all problems due to the absence of training using large datasets. Hence, future research may use the bigger datasets for the problem or even create a standard feedstock library for investigation in this area, especially where ML techniques are still in their infancy.

#### Others

In this subsection, the other quantitative approaches for facing uncertainties in the BSC problem are discussed, most of which devotes to hybrid techniques. One notable advantage of hybrid techniques in BSCR is their ability to compensate for the limitations of one method with the strengths of another. Ren et al. (2016) developed an interval mathematical model to optimize the BSC by minimizing lifecycle energy and CO_2_ emission. They defined uncertainty in feedstock availability, transportation capacities, and demand as confidence intervals. A risk-sharing model was studied by Ye et al. (2018) for coordinating the players’ decisions in a cassava-based BSC under uncertainty of feedstock yield and biofuel demand. Since variability in biomass availability is considered one of the most common sources of uncertainty, Santibañez-Aguilar et al. (2018) proposed a framework based on a geographic information system (GIS) to find suitable facility sites for supply networks according to residual feedstock. A two-stage supply chain model for solid biomass fuel was studied by Fan et al. (2019), which included designing two contracts between the manufacturer and farmers as well as the manufacture and middleman in the network under supply and demand uncertainty. To face uncertainty in feedstock availability, Martinkus et al. (2017) proposed two past-predictive and future-predictive methodologies to analyse and predict the quantity and cost of forest-based feedstock supplied to a biorefinery plant according to the available data.

Since each quantitative approach has various capabilities, various quantitative approaches have different degrees of application to the different stages of the BSCR problem, as seen by this research. By way of illustration, consider mathematical models that are effective in preparedness and response stages, but they cannot make automated decisions and handle large data. However, these can be achieved using ML methods that are less successful in modelling complex BSC networks. Consequently, researchers have investigated the hybrid technique to address basic quantitative approaches’ limitations.

Azadeh and Arani (2016) studied an integrated approach, including system dynamics and mathematical modelling, to develop a BSC network from farms to consumers. Their methodology employed the system dynamics to estimate the required parameters used as the input of the stochastic mathematical model. A hybrid approach constituting the simulation and mathematical modelling techniques was developed by Ebadian et al. (2014), where the simulation phase utilizes the tactical decisions provided by the optimization model to obtain operational decisions for supplying feedstock to the bioethanol production plant. In an alternative hybrid approach, Höltinger et al. (2014) introduced a mathematical model to optimize the production of a green biorefinery by determining optimal locations and sizes for the facilities involved. Then, they employed the Monte Carlo simulation to investigate the effect of input parameters (mainly the product prices) on the obtained results. Hong et al. (2014) investigated a robust optimization model based on the simulation technique to determine the location of biofuel facilities and transportation decisions in a bio-energy logistics network when biomass yield acts as the uncertain parameter. To incorporate different types of uncertainty in a rice straw-based BSC, Diehlmann et al. (2019) proposed a hybrid simulation–optimization framework where technological and economic uncertainties were involved in a Monte Carlo simulation. Then a two-stage stochastic optimization model was used to determine the facility location and transship decisions by considering political, demand, and price uncertainties. Ngan et al. (2020) believed that conventional risk mitigation strategies, such as stochastic optimization, could not incorporate non-quantitative risks into the problem. Consequently, they devised a hybrid technique that combines an analytical model with stochastic optimization to assess and mitigate risks within biofuel production systems.

## Discussion of current research gaps and future research directions

The preceding section highlighted the notable capacity of quantitative techniques to handle uncertainties and disruptions in BSC problems. However, there are still critical gaps that require further investigation in this area. The remainder of this section will introduce and suggest some of these research directions.

### Lack of realistic assumptions

One of the significant findings of this study is that existing research in BSC has focused more on purely theoretical aspects rather than solving real-world challenges. However, it is crucial for researchers to consider how they can incorporate realistic assumptions into their problem models. An analysis of the reviewed papers reveals that 96.6% primarily examined networks with forward flow, while only 3.4% considered both backward and forward flows. However, incorporating backward flow in the models can bring them closer to real-world circumstances, as it reflects actual processes and practices observed in BSCs. For example, the organic fertilizers can be sent back to the biomass supply regions due to biomass residuals produced in conversion plants (Balaman and Selim 2014b; Mohseni and Pishvaee 2016) or extraction sites (Babazadeh 2019). Also, the by-products provided by biorefineries may be reprocessed and used in other facilities (De Meyer et al. 2015; Garai and Sarkar 2022). These examples reflect the actual processes and practices that occur in real-world BSCs but have been overlooked in studies.

The findings of our study reveal that the concept of the ripple effect, which signifies the propagation of disruptions throughout the supply chain network, has received limited attention within the BSC domain. Surprisingly, only 3.4% of the shortlisted papers incorporated this concept, with only two papers utilizing simulation and network-based techniques. These statistics underscore the considerable potential of mathematical modelling and machine learning approaches as prescriptive and descriptive tools for studying BSCs in the presence of ripple effects. The study also confirms that the ripple effect is an essential consideration in almost all types of supply chains, regardless of their complexity, as disruptions cannot be localized and have network-wide consequences (Habibi 2022). By addressing this research gap and employing advanced methodologies, researchers can gain deeper insights into BSC dynamics and effectively mitigate the impact of ripple effects.

### Neglected uncertainty sources

The findings of our study shed light on the multitude of uncertain parameters that play a role in BSC problems, as demonstrated in Table 2. An essential step in mitigating the impacts of such uncertainties is identifying the most impactful sources of uncertainty and devising effective solutions accordingly. Table 2 presents compelling evidence, indicating that feedstock availability, final product demand, and biofuel price are the most prevalent uncertain parameters, accounting for 58%, 41.5%, and 20.5%, respectively, as investigated by the reviewed papers. Conversely, certain parameters such as workforce availability, market structure, losses due to transportation, return percentage, transport distance, economic impact, carbon tax rate, recycling cost, technical factors, harvest rate, import price, and transportation capacity have received comparatively less attention but hold potential for future research exploration. Notably, some influential parameters that significantly impact BSCs have been overlooked in the related literature. Some of them are safety-stock inventory in sites (Shi and You 2022), production time (Pasandideh et al. 2015), the disposal rate of returns in a backward network (Subulan et al. 2015), the buying price of returns in a backward network (Liao et al. 2022), and the profit of returned products (Jindal and Sangwan 2014). While the importance of these uncertain parameters has been highlighted in studies, their exploration within the context of BSC problems has remained largely uncharted territory.

### Shortcomings in uncertainty modelling

**Fig. 5:**
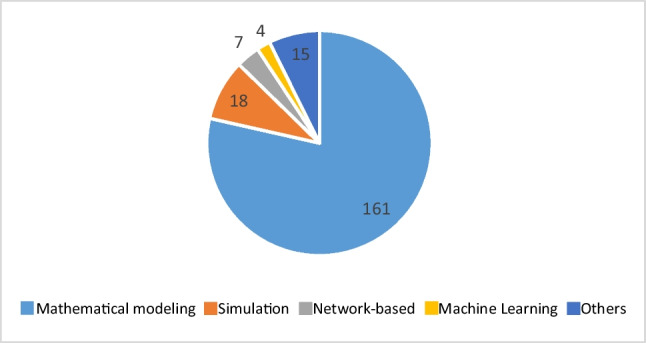
The number of reviewed papers in terms of their methodologies

This study shows that each methodology in the BSCR domain serves a specific purpose and yields distinct outcomes, resulting in varying utilization levels. The findings of our research and Fig. 5 reveal that mathematical modelling has been the predominant choice, accounting for 50% of the reviewed papers, while other quantitative approaches have received less attention. Machine learning, network-based, and simulation techniques, with respective shares of 2%, 3%, and 9%, have not been extensively explored in the context of BSCR despite their proven effectiveness. The preference for mathematical modelling can be attributed to its exceptional precision and well-established rules, enabling decision-making with a high level of accuracy and accommodating various assumptions. Moreover, its long history, dating back to the 1960s, may have contributed to its wider adoption compared to relatively newer methods like machine learning. However, it is essential to acknowledge the potential of these alternative approaches, which have demonstrated their efficacy in other domains. In the subsequent sections, we discuss the specific research gaps identified within each methodology, highlighting the need for further investigation and exploration. This analysis will provide valuable insights into areas where additional investment and attention are warranted for future research in the BSCR field.

#### Mathematical modelling

**Fig. 6:**
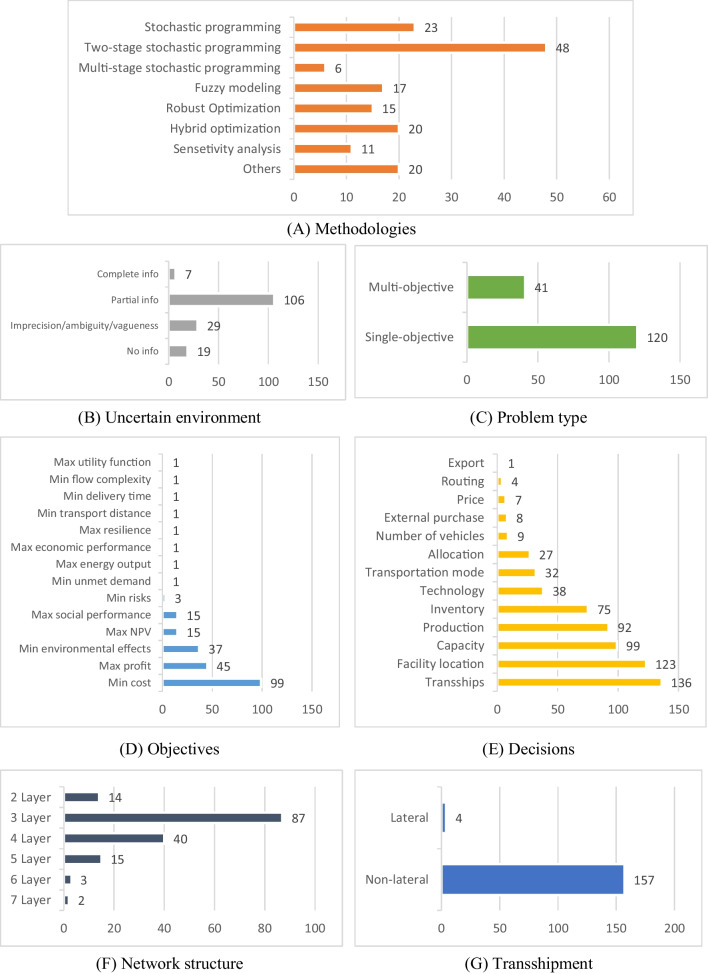
Statistics regarding the shortlisted papers studied mathematical modelling

The results of the literature review reveal that mathematical modelling has been extensively explored, primarily due to its ability to provide optimal decisions for designing the BSC network. However, there remain key areas that warrant further investigation based on our findings. Figure 6 illustrates the distribution of papers investigating different mathematical modelling features, offering valuable insights into potential research directions. Observing Fig. 6A, it is evident that stochastic programming approaches have been the most commonly employed techniques for modelling uncertainties in BSCR problems. However, relatively newer avenues such as fuzzy modelling, robust optimization, and hybrid approaches present promising areas for exploration, particularly in scenarios where parameter information is scarce or where imprecision, ambiguity, and vagueness are present (see Fig. 6B). Figure 6C reveals that a majority (approximately 75%) of the papers focused on modelling the BSC problem as a single-objective optimization. However, considering the conflicting objectives within a BSC system as a multi-objective problem opens up avenues for further study. Additionally, Fig. 6D indicates that economic objectives, such as cost minimization and profit maximization, have received greater attention compared to objectives like service time minimization, transport distance minimization, resilience maximization, and unmet demand minimization. Notably, objectives such as reliability maximization, robustness maximization, and disruption cost minimization remain relatively unexplored, offering the potential for future investigations.

Examining the major decisions in the BSC network (Fig. 6 E), it is apparent that certain aspects, such as external sales, selling price, external purchase, and the number of required vehicles, have received less attention compared to others. Furthermore, decisions pertaining to resilience strategies, including selecting backup suppliers, multisource allocation, and multi-communication paths, have been largely overlooked. Incorporating these critical decisions can enhance the practicality of BSC problems for real-world applications. Regarding network structure (Fig. 6F), the three-layer structure comprising supply, processing, and demand points has been extensively studied, while networks with six or seven layers have received less consideration due to their inherent complexity. However, leveraging decomposition and metaheuristic algorithms can help tackle the complexity associated with these multilayer networks. Moreover, incorporating lateral transshipments can better align the problem with real-world circumstances and enhance network resilience, a concept that has been rarely explored, as depicted in Fig. 6G. Based on these findings, several research questions emerge for future studies:

- How can non-linear programming techniques address the modelling challenges posed by the complexity of real-world BSC problems, which often surpass the capabilities of simple linear optimization techniques?
- How can the interests and benefits of different stakeholders, including government entities, public organizations, and private entities, be considered in BSC decision-making? Developing a multilevel optimization model could address this challenge effectively.
- What approaches can be proposed to develop a decision support system that incorporates optimal decision-making across pre-disaster, disaster, and post-disaster periods, particularly when strategic, tactical, and operational decisions are integral to the decision-making process?

These research questions highlight the potential areas for future exploration and provide a roadmap for advancing the field of mathematical modelling in BSCR.

#### Simulation

**Fig. 7:**
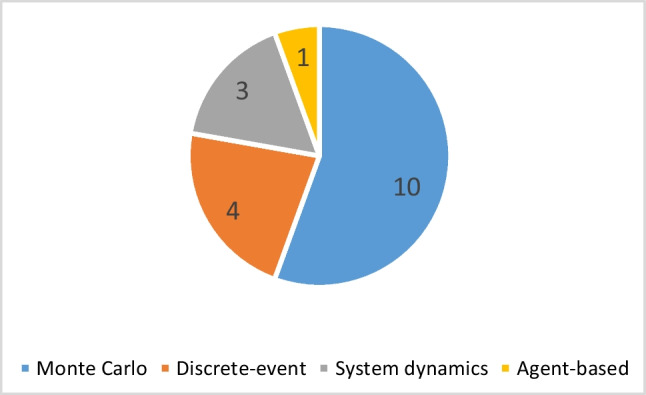
The number of reviewed papers studied simulation approaches

Drawing upon the findings of our study, it is evident that there is significant potential for further development of simulation techniques within the BSCR domain. Figure 7 unveils notable trends in simulation methodologies, where more than half of the research focuses on Monte Carlo simulations, followed by discrete-event and system dynamics. However, agent-based simulation, an emerging modelling approach with the ability to effectively analyse agent actions and interaction networks, exhibits significant potential for future research exploration. The application of agent-based simulation in BSC can be extended to various areas, including evaluating the efficiency of mitigation strategies against disruptions (Lu et al. 2021), evaluating resilience (Aghababaei and Koliou 2022), predicting uncertain parameters (Achmad et al. 2021), and investigating the influence of blockchain technology on supply chain resilience (Li et al. 2022; Lohmer et al. 2020). Besides, the influence of network structure on BSCR, an important aspect, has been overlooked thus far. Simulation techniques offer an appropriate avenue for investigating how network types, characteristics, disruptions in nodes and links, and ripple effects influence biofuel supply dynamics. Their ability to handle complexities makes simulation techniques ideal for analysing large-scale problems where mathematical models may fall short. Based on our study’s findings, we propose several research questions for future investigations in this area:

- How can risk-based properties, such as robustness, agility, and resiliency, be estimated in BSCs?
- What are the effects of proactive and reactive mitigation policies on the resiliency of BSCs?
- How can disruption propagation be effectively managed when uncertainties result in the disruption of entities in the BSC?
- What are the interaction effects of uncertainties when the BSC faces multiple sources of uncertainties?

These research questions, derived from our study’s insights, provide valuable directions for future research endeavours, facilitating the exploration and advancement of simulation techniques in the context of BSCR.

#### Network-based techniques

**Fig. 8:**
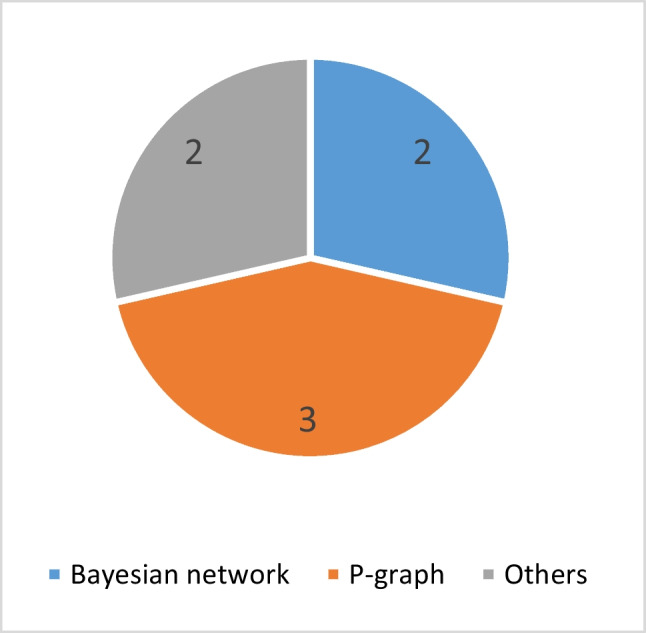
The number of reviewed papers studied network-based approaches

Based on our study’s findings, it becomes apparent that there exists substantial untapped potential for advancing simulation techniques in the context of the BSCR domain. Figure 8 reveals that the utilization of methods within this category is relatively low, with only two and three papers studying Bayesian network and P-graph techniques, respectively. However, several avenues remain unexplored, presenting opportunities for future research. In the context of BSCR, Petri net models can be utilized for risk identification and management (Liu et al. 2018; Wang et al. 2022), allowing for the investigation of the effects of disruptions in links or nodes on the network. Additionally, the application of a graph neural network (GNN) holds promise in efficiently detecting hidden relationships and extracting information from graphs (Liang et al. 2022). This tool facilitates a comprehensive understanding of interdependencies among different facilities in the BSC, thereby enhancing visibility into the risks they face. Based on our study’s findings, we propose several research questions to be explored in future studies:

- How can non-linear relationships between different uncertainty sources in BSCs be interpreted? Bayesian network possesses the ability to interpret such relationships effectively.
- How can BSC resilience be measured by considering the interdependencies among the resilience drivers implemented within the system? Network-based techniques, such as the graph theory matrix approach, offer a framework for measuring these interdependencies.
- In today’s business world, characterized by abundant uncertainties, are there any hidden uncertainty sources within BSCs? In such cases, fault tree analysis can be employed to identify new sources within complex BSC structures.

These research questions, grounded in the findings of our study, present promising avenues for future investigations. By exploring network-based techniques, researchers can expand their understanding of the dynamics of BSCR and develop robust frameworks to address uncertainties and enhance overall system resilience.

#### Machine learning techniques

Based on the findings of our study, it is evident that machine learning (ML) approaches have received relatively less attention within the BSCR field, despite their numerous merits. Our analysis identified a total of four papers on ML techniques, including two focused on prediction techniques and two exploring ML applications in other fields. The “Machine learning approaches” section delved into the limitations of these approaches and discussed future directions to address those limitations. The significant developments in the ML domain present a compelling opportunity to employ various ML techniques in managing uncertainties and disruptions within BSC networks. For instance, utilizing classification and clustering techniques like decision tree (DT) and support vector machine (SVM) methods for risk identification, reinforcement learning for resilient supplier selection, big data analytics for risk assessment, and long short-term memory (LSTM) for demand prediction are potential avenues for applying ML models in BSC. It is worth noting that most ML approaches are designed to work with predetermined problem sets, where both training and validating data originate from the same statistical distribution. However, these techniques face limitations when real-world data violate the assumed statistical distributions. Exploring adversarial machine learning strategies can address this limitation within the BSCR context. Additionally, reinforcement learning emerges as another suggestion that can support managers in proactively identifying operational risks through autonomous learning and appropriate action (Aboutorab et al. 2022). As AI techniques can significantly impact performance within the BSC network, decision-makers must understand how AI models operate and reach decisions. However, the investigation of Explainable AI (XAI) methods, which interpret models to humans for efficient and appropriate decision-making (Holzinger et al. 2022), remains unexplored in the BSCR field. Employing XAI methodologies can yield several significant benefits, including reducing the likelihood of decision-making errors, identifying potential model weaknesses for improvement, and determining key drivers for effective decision-making.

In light of these findings, several potential research questions emerge:

- How can ML techniques be utilized to better estimate uncertain parameters based on historical data, particularly in conjunction with other methodologies such as stochastic optimization or fuzzy modelling?
- How can real-world data be leveraged to provide managerial insights for managing uncertainties and disruptions in BSCs? For example, identifying the most frequent and impactful sources of uncertainties in real-world BSCs or determining the most effective mitigation strategies under real-world circumstances.
- What early warning or real-time monitoring systems can efficiently minimize the detrimental effects of uncertainties in BSCs? ML techniques can be employed to propose and develop such systems.

These research questions, informed by our study’s findings, offer valuable pathways for future investigations.

### Other novel areas

Drawing on the findings of our study, it is evident that hybridization, particularly the combination of machine learning (ML) and mathematical modelling, has not received the attention it deserves among other quantitative techniques. This approach offers the potential to overcome the weaknesses of individual methods. In contrast to the widely used sensitivity analysis, employing counterfactual analysis can provide insights into the impact of government interventions and actions on efficiency within the BSC network (Levi et al. 2021). Policies and regulations not only serve as significant sources of uncertainty but also exert a substantial influence on actions in the BSC network. The Belief Rule Base (BRB) approach, functioning as an expert system, provides a framework for capturing uncertain data using knowledge representation. This approach can be applied when there is uncertainty in biofuel demand or production capacity influenced by other parameters that may not conform to assumed statistical distributions (Zhao et al. 2022). Furthermore, our study reveals that the majority of reviewed papers have focused on enhancing BSC performance against uncertainties and disruptions. However, an essential initial step towards improvement entails evaluating the current state of the system and making appropriate decisions based on identified weaknesses. Therefore, there is a clear need to develop a resilience evaluation framework that can identify the specific requirements of the system.

By considering these insights, the following potential research directions can be explored:

- How can hybridization techniques, such as combining ML and mathematical modelling, be effectively utilized to enhance decision-making and address uncertainties in the BSC domain?
- What are the key factors and methodologies involved in conducting counterfactual analysis to study the impact of government interventions and actions on BSC efficiency?
- How can the Belief Rule Base (BRB) approach be applied to capture and manage uncertain data, particularly in cases where biofuel demand is influenced by various parameters deviating from assumed statistical distributions?
- What are the essential components of a resilience evaluation framework for BSCs, and how can it effectively identify and address system needs?

These research questions, rooted in our study’s findings, provide valuable directions for future research endeavours.

## Conclusion

In today’s uncertain world, the complexity and challenges associated with supply chain planning are amplified. This emphasizes the significance of developing methodologies to effectively address uncertainties within the supply chain network, particularly in the context of biofuel supply chains (BSCs) as a pivotal renewable energy source. However, existing review studies on uncertainties in BSC have certain shortcomings. Some studies have a limited scope, focusing only on specific types of biofuel generation. Certain reviews lack a comprehensive examination of uncertain sources and methodologies, while others are outdated and do not consider recent advancements. Additionally, some book chapters touch on uncertainties and modelling approaches but fail to thoroughly analyse limitations or provide specific suggestions for future research. The purpose of this review paper was to overcome these limitations by consolidating the existing frameworks, identifying their limitations, and presenting the necessary advancements to aid researchers in this area. The study followed the rigorous Systematic Reviews and Meta-Analyses (PRISMA) methodology, which enabled the identification of 205 relevant papers for analysis. Through meticulous classification and analysis of the theories and methodologies employed in these papers, we effectively addressed the first research question pertaining to the involvement of uncertainties in managing BSCs. Furthermore, our analysis successfully addressed the second research question posed in this paper by illuminating the limitations and shortcomings of current techniques in effectively managing uncertainties within BSCs. Despite the significant efforts that have been made in this area, it is evident that there are still important aspects that warrant further investigation and improvement.

### Summary of recommendations

The findings of this study highlighted the need for continued research and innovation to overcome the identified shortcomings and advance the field of uncertainty management in BSCs. To address the third research question in this study, we have provided a wide range of new directions and research avenues that hold promise for addressing these challenges effectively. Here is the summary of the critical potential directions that can be followed in future research:

- Backward flow, lateral transshipment, and ripple effect are valuable concepts, all of which are needed to be considered when studying BSC. Backward flow analysis helps optimize resource utilization and minimize biofuel waste by examining the reverse movement of biomass residuals produced in conversion plants within the chain,  for example. Lateral transshipment facilitates efficient distribution among facilities within the same tier, such as biofuel production centres. Considering the ripple effect enables the identification of vulnerabilities and the development of strategies to mitigate risks and enhance resilience throughout the BSC. For instance, decision-makers can assess how disruptions in biomass production or pre-treatment centres may impact the overall service level. Understanding these concepts provides a solid foundation for the development of effective policies that promote sustainability and efficiency in the BSC.
- The BSC presents various sources that require further discussion and solutions. Workforce availability (e.g. skilled labour shortages in biofuel production centres), market structure (e.g. fluctuations in biofuel demand due to government policies), transportation capacity, losses due to transportation, return percentage (e.g. biofuel returns due to quality issues), transport distance, economic impact, carbon tax rate, recycling cost, technical factors (e.g. conversion rate), harvest rate, and import price are crucial factors that warrant in-depth examination. Addressing these factors can help optimize workforce management, improve market dynamics, enhance transportation efficiency, reduce losses during transportation, optimize return management, minimize environmental impacts, and optimize economic performance. Additionally, certain uncertainty sources have not been thoroughly studied in BSC papers, including safety-stock inventory in sites, production time, the disposal rate of returns in a backward network, the buying price of returns in a backward network, and the profit of returned products. Exploring and understanding these uncertainty sources lead to recommendations for better management, adjustments, optimization, and the adoption of advanced technologies to enhance overall performance.
- While proposing mathematical models for BSCs, objectives such as reliability maximization, robustness maximization, and disruption cost minimization can be modelled. Furthermore, exploring decisions related to implementing resilience strategies, such as selecting backup suppliers, multisource allocation (e.g. multi suppliers for biofuel production centres), and multi-communication paths (e.g. utilizing multiple transportation methods like road, rail, and pipeline for biofuel transfer), can enhance the overall resilience of the BSC. By understanding and implementing these concepts, researchers can propose specific policy recommendations that promote resilience, efficiency, and sustainability in the BSC, considering the challenges of the decision-makers.
- Agent-based simulation is an effective approach that can be further applied to study the BSC. For example, researchers can use agent-based simulation to analyse the efficiency of mitigation strategies against disruptions, such as simulating the impact of a sudden feedstock shortage or transportation disruption on the overall BSC performance. This approach can also evaluate the BSC resilience by simulating various scenarios and assessing how the system adapts to disruptions. Furthermore, agent-based simulation can be used to predict uncertain parameters, such as biofuel demand fluctuations, and investigate the influence of blockchain technology on BSC resilience by simulating the implementation of blockchain-based traceability and transparency solutions. By employing agent-based simulation, researchers can gain valuable insights into the BSC dynamics, enabling them to make informed decisions and develop strategies that enhance resilience and optimize performance.
- Network-based approaches offer valuable insights into studying risks and uncertainties within the BSC. For example, researchers can utilize Petri net models to analyse the risk of feedstock availability. By simulating different scenarios, they can assess the impact of uncertainties such as crop failures, weather events, or changes in agricultural practices on biofuel production and distribution. Additionally, employing graph neural networks (GNN) or Bayesian networks can help detect relationships among uncertainty sources, such as studying the effects of transportation disruptions on the BSC. By analysing factors like road closures, port congestion, or shifts in transportation modes, researchers can assess the implications of delivery delays, increased costs, and potential bottlenecks. Furthermore, network-based approaches enable the analysis of market demand fluctuations, allowing researchers to investigate the relationship between factors like consumer behaviour, government policies, and biofuel market dynamics. This analysis aids in understanding the uncertainties associated with market demand and developing strategies to adapt to changing conditions within the BSC.
- Machine learning techniques can offer a range of potential applications in the BSC domain. For example, classification and clustering methods, such as decision tree (DT) and support vector machine (SVM), can be utilized to identify and classify risks within the BSC. These models can help analyse data related to feedstock availability, transportation disruptions, or market demand fluctuations, enabling proactive risk identification and mitigation. Reinforcement learning algorithms can facilitate resilient supplier selection by training models on historical data to identify suppliers that demonstrate adaptability and resilience in the face of uncertainties, such as price fluctuations or delivery disruptions. Furthermore, big data analytics can be employed for comprehensive risk assessment in the BSC, utilizing large-scale data sources to identify patterns and correlations that contribute to risk exposure. For instance, market trends, environmental factors, and economic indicators can be analysed to develop strategies that mitigate potential disruptions. Additionally, long short-term memory (LSTM), a type of recurrent neural network, can enhance demand prediction accuracy by incorporating relevant variables, such as seasonality, market dynamics, and macroeconomic factors, leading to improved production planning, inventory management, and overall supply chain optimization. By harnessing the capabilities of machine learning techniques, researchers can enhance risk management, optimize decision-making, and bolster the resilience and efficiency of the BSC.

### Study limitations

The current study focused primarily on quantitative approaches for managing uncertainties in the BSC, thereby excluding empirical studies that used qualitative techniques. While this exclusion may limit the comprehensive understanding of uncertainties in BSCs, it highlights an important direction for future research. Prospective scholars can review qualitative techniques, such as empirical studies that used questionnaires or interviews, to capture subjective experiences, stakeholder perspectives, and nuanced insights on uncertainties in BSCs. By incorporating qualitative approaches, researchers can enhance the understanding of uncertainties from a broader perspective and provide a more holistic analysis of the challenges and potential solutions in managing uncertainties in BSCs. An additional limitation of this study was the omission of a detailed examination of the effects of COVID-19 on BSC networks. Given the significant impact of this global crisis, it warrants extensive investigation that goes beyond the scope of this study. Understanding the lessons learned from the COVID-19 disruption is crucial for maintaining the resilience and health of BSCs in potential future crises. Therefore, it is highly recommended that future research invests considerable effort into examining the effects of COVID-19 on BSC networks to inform strategies for crisis management and enhance the overall resilience of the biofuel supply chain. This review paper did not include an in-depth analysis of case studies investigating uncertainty management in the BSC, limiting insights into real-world applications and practical challenges. Future research should focus on conducting a comprehensive review of case studies to analyse their methodologies, key findings, and lessons learned. This would provide researchers with practical insights and inform decision-makers in effectively managing uncertainties in the BSC.

## Figures and Tables

**Table 3:**
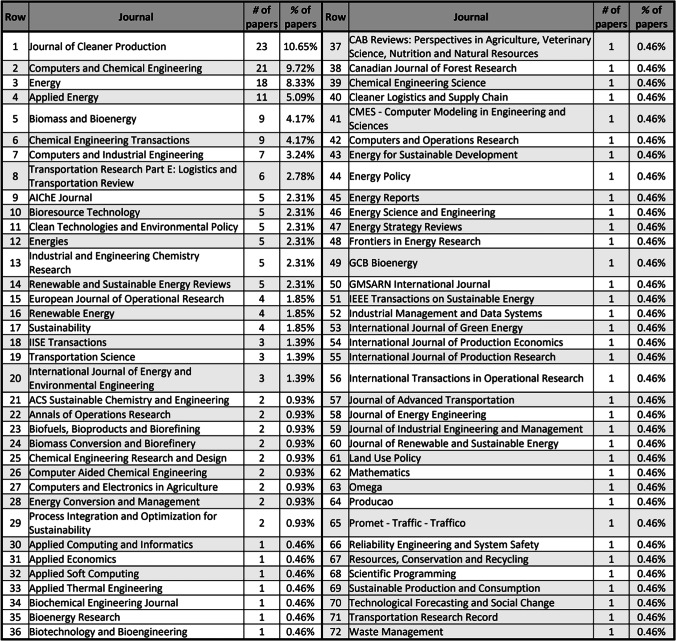
The submission and publication years of shorlisted papers

## Data Availability

The authors confirm that the data supporting the findings of this study are available within the article.

## Appendix

See Table 3
